# A window-DEA based efficiency evaluation of the public hospital sector in Greece during the 5-year economic crisis

**DOI:** 10.1371/journal.pone.0177946

**Published:** 2017-05-23

**Authors:** Angeliki Flokou, Vassilis Aletras, Dimitris Niakas

**Affiliations:** 1 School of Social Sciences, Hellenic Open University, Patra, Greece; 2 Department of Business Administration, University of Macedonia, Thessaloniki, Greece; Kingston University, UNITED KINGDOM

## Abstract

The main objective of this study was to apply the non-parametric method of Data Envelopment Analysis (DEA) to measure the efficiency of Greek NHS hospitals between 2009–2013. Hospitals were divided into four separate groups with common characteristics which allowed comparisons to be carried out in the context of increased homogeneity. The window-DEA method was chosen since it leads to increased discrimination on the results especially when applied to small samples and it enables year-by-year comparisons of the results. Three inputs -hospital beds, physicians and other health professionals- and three outputs—hospitalized cases, surgeries and outpatient visits- were chosen as production variables in an input-oriented 2-year window DEA model for the assessment of technical and scale efficiency as well as for the identification of returns to scale. The Malmquist productivity index together with its components (i.e. pure technical efficiency change, scale efficiency change and technological scale) were also calculated in order to analyze the sources of productivity change between the first and last year of the study period. In the context of window analysis, the study identified the individual efficiency trends together with “all-windows” best and worst performers and revealed that a high level of technical and scale efficiency was maintained over the entire 5-year period. Similarly, the relevant findings of Malmquist productivity index analysis showed that both scale and pure technical efficiency were improved in 2013 whilst technological change was found to be in favor of the two groups with the largest hospitals.

## Introduction

The effects of the 2007–08 financial crisis were strongly felt in Greece in the years that followed. In 2013, the economy entered the sixth year of recession, resulting in a substantial GDP decline. The main impact of the economic crisis was on the unemployment rate which rose eighteen percentage points from 9.6% (485,000 persons) in 2009 to 27.5% (1,330,000) in 2013 [1]. The main share of jobless workers was from the private sector which amounted to 769,000 lost jobs in the years 2008–2012 compared to 89,000 in the public sector. At the same time, the sharp rise in the unemployment rate led to an equally sharp increase in poverty as the percentage of the population that was below the poverty threshold in 2012 increased to 38% [2]. It is notable that in a survey from the Hellenic Statistical Authority (ELSTAT) [1] the relative index of “people at-risk-of-poverty or social exclusion” in 2013 (after five years of austerity) was higher by 8.1 percentage points compared to 2009 (35.7% vs 27.6%) whereas the corresponding increment in Eurozone (EU-28) was only +1.3 pp (24.5% in 2013 vs 23.2% in 2009). In the same survey, the rate of “severe material deprivation” in 2013 was also shown to have climbed by 14.3 percentage points since 2009 (37.3% vs 23.0%).

The recession hit primarily the younger generation as was indicated by the share of young people in the unemployment index which amounted to almost 49%. The dramatic increase in youth unemployment and the 'scarring' effects of joblessness generated a large wave of human outflow from the country, mainly among educated and qualified people, scientists and other professionals in foreign countries. It is worth noting that according to the Athens Medical Association, there was a fivefold increase in the number of skilled Greek physicians who migrated abroad in 2012 compared to 2007 (1,166 vs 292 doctors respectively) [3–4]. The overall emigration showed an increasing trend and almost tripled from 2009 to 2013 (43,686 vs 117,094 people respectively) [5].

At the same time, an additional side effect of people’s inability to cover their insurance contributions because of unemployment and the undeclared work was the loss of their insurance coverage (and family dependents). It is worth noting that between 2008–2012, one out of three insured members in the two largest insurance organizations (IKA and OAEE) lost their health insurance eligibility [2]. The large increment in the number of uninsured citizens has in turn resulted in limited or no access to medical care and pharmaceuticals exacerbating the inequalities in health care provision and increasing out-of-pocket expenses [6]. It is notable that the share of household payments to public hospitals over the total household health expenditure rose substantially by 86% in the four-year period 2008–2012 (4.2% vs 7.8%) [7].

During the same period, there has also been a deterioration in the mental health of the population which has been attributed -directly or indirectly- to the economic crisis and high unemployment [8]. The incidence of major depression increased by nearly five percentage points (from 3.3% to 8.2%), especially among young people [9]. Other studies have recorded a 35% increase in the number of suicides (from 3.37 to 4.56 per 100,000 of the population between 2010 and 2012) [10], as well as in the number of people who had attempted suicide, with those who were experiencing financial difficulties to be in a particularly vulnerable group [11]. The number of reported violent incidents also increased while the rate of homicide and theft cases almost doubled between 2007 and 2009 [12–13].

Together with the deterioration of mental health there was also evidence for the worsening of general health, particularly in vulnerable groups, as reported by Kentikelenis et al. [12]. As for the perceived health status, the percentage of people who assessed their health at the level of "good" to "very good" decreased from 75.5% in 2009 to 74.1% in 2013, whereas the corresponding proportion for the assessment at the level of "bad" to "very bad" increased from 9.7% to 10.4% during the same period [1]. A study from Zavras et al. [14] verified relevant findings from previous studies which have shown that better levels of self-rated health are positively associated with income and education, the two variables that mostly characterize the socio-economic status of the population. At the same time the association was also found -as expected- to be negative with unemployment, the existence of chronic disease and age. In the same vein another study of Yfantopoulos et al. [15] reported that the economic crisis has also led to an aggravated level of general and oral health in Greece. The study identified statistically significant oral health inequalities among the socio-economic groups with the aged, the less educated and those confronting financial difficulties to be associated with lower levels of oral health.

In the same period, an HIV epidemic outbreak was also recorded among heroin addicts owed mainly to budget cuts that led to the cancellation of many preventive programs (exchange of needles etc.) [12–13]. However, it should be noted that although drug use increased between the years 2007–2011, the per capita alcohol consumption in the same period decreased by 2.3% [13].

As far as mortality is concerned, the relevant data published by ELSTAT [1] showed that the number of deaths increased from 108,316 in 2009 to 111,794 in 2013. It is notable that the 116,670 deaths that occurred in 2012 was the largest recorded number since 1949 [16] whilst 2,000 of them (almost one third of the additional number of deaths) could be linked to austerity. However, it should be noted that the total number of dead and injured in road accidents has followed a continuously downward trend (20,097 in 2009 vs 16,054 in 2013) [1]. The life expectancy in the same period continued an upward trend which had been recorded in the pre-crisis years. Specifically, according to ELSTAT [1], the relevant index increased from 77.7 years in 2009 to 78.3 years in 2013 for men and from 82.8 to 83.4 years for women, amounting to average annual increments of 0.27 and 0.20 years respectively.

In 2013, the GDP declined by almost one quarter compared to 2007 whilst in the same period, health care spending followed a parallel trajectory. It is noteworthy that in the years from 2005 to 2009, where GDP increased by 19.2%, the corresponding total health spending rose by 41% [17]. The positive sign of the slope was reversed after the onset of the crisis. In the years between 2009–2012, the GDP declined by 16.4% which was accompanied by a 23.1% downturn in the total health care expenditure and 24.5% reduction in the public health spending (from 23.0 to 17.7 and from 16.1 to 12.0 billion € respectively) [18].

In terms of GDP percentage, total health care expenditure was increasing until it reached 9.9% of GDP in 2009 where it started declining to reach 9.1% of GDP in 2012. In the same period the GDP percentage of public health care expenditure was reduced from 7% in 2009 to 6.2% in 2012. Within this period and in order to fulfill loan conditions which require that public health care expenditures must not exceed 6% of GDP, The Ministry of Health had to go through a wide range of reforms aiming, among others, at the following goals: to achieve greater efficiency in all NHS services and, at the same time, to achieve a radical cut in expenses.

Specifically, the reform efforts were focused on two targets. The first was to merge neighboring public hospitals, as well as hospital clinics and departments, and the second to modernize the financing mechanism used by the unified, state-owned health insurer (EOPYY), by introducing a Greek version of Diagnostic Related Groups (DRGs [19]). Regarding the first reform, with the exception of 2–3 small hospitals which were integrated into larger units, hospital mergers were never implemented in Greece due to extensive political and social pressure by respective interest groups. As for the intended plan for EOPYY to finance public hospitals via the DRG system (known as KEN-DRGs in Greece), this is pending on the unlikely event that EOPYY can solve its inherent deficit problems and find sufficient resources to fulfill its intended purpose. Hence, public hospitals continue to be funded by The Ministry of Health through the state budget, which covers salaries and all current expenses. Apart from closed budgets, two horizontal measures were adopted by the Ministry to reduce public health care expenditures. One measure was an average 35% cutback in salaries since 2009, and the other an “until further notice” suspension of all public hiring. In light of these developments, the aim of this study was to investigate longitudinal efficiency of Greek public hospitals during the crisis period.

### The hospital sector of Greek National Health System in the years of crisis

The Greek National Health System (NHS) includes 131 (general, special, university and university-affiliated) hospitals which admit more than 2.2 million patients per year. All NHS hospitals are geographically partitioned in seven administrative regional units. Each regional unit serves as the management link -at an intermediate level- between individual hospitals and the Ministry of Health. These hospitals constitute the backbone of the Greek NHS as they provide the bulk of secondary, tertiary as well as primary health care services to the majority of the population. Table 1 reports a summary of operational characteristics of Greek NHS hospitals. As it can be seen, there was a substantial increase in the demand for health care services from public hospitals as evidenced by the relevant increments in the total numbers of hospitalized cases (32.9%) and surgeries (9.2%) during the period 2009–2013. The increased demand for health care services in the public sector was due to a shift of patients from the private sector, mainly of lower income. In the 2009–2010 period, a decrease had been recorded in the total number of admissions in private hospitals by 25–30%, owed mainly to the inability of lower income groups to afford the relative high costs [6]. In addition, increased demand for dental and obstetric services in public hospitals was also recorded, two areas that had been traditionally covered by private providers/hospitals up until then [20].

**Table 1:** Greek NHS data.

	2009	2010	2011	2012	2013
Beds	33,693	34,917	34,577	35,020	34,092
Physicians	22,084	22,056	20,074	20,824	18,853
Other staff	64,135	68,429	62,609	61,742	57,934
Inpatient days	8,128,922	8,867,428	9,166,881	9,080,808	8,782,234
Inpatient cases	1,711,352	2,118,868	2,218,465	2,284,316	2,273,751
Outpatient visits	13,056,652	11,911,390	11,797,396	11,794,499	11,883,538
Surgical operations	417,843	436,405	461,677	456,897	456,364
Laboratory tests	145,639,726	129,584,563	153,154,593	152,531,500	133,239,959
ALoS	4.75	4.18	4.13	3.98	3.86
Occupancy rate	0.66	0.70	0.73	0.71	0.71
Total NHS Expediture (mio €)	2,751.37	2,614.91	2,443.26	1,490.06	1,323.52

At the same time, there was a substantial gradual reduction in the average length of stay (ALoS) from 4.75 days in 2009 to 3.86 days in 2013 (i.e. -18.7%). The increased number of admissions combined with the reduced mean length of stay accounts for an overall reduction of 8% in the total number of inpatient days for the same five-year period. It is also interesting to note that there was an 8.5% reduction in the number of laboratory tests between 2009 and 2013 after an intermediate peak of 5.1% which occurred in 2011. It should also be taken into account that since the onset of the crisis, certain nongovernment organizations such as Médecins du Monde and Medecins Sans Frontieres, which had been providing health care services mainly to immigrants, started to cover additional groups of the population including the poor, the unemployed and the uninsured [20].

According to ELSTAT [1], the percentage of persons with self-declared unmet needs for medical examination or treatment (due to several reasons including financial barriers, long waiting times and traveling distances, lack of time etc.) increased from 4.1% in 2009 to 11.2% in 2013. Taking into account that all citizens could visit almost at no charge either general practitioners or outpatient clinics in hospitals, Kentikelenis et al. [12] concluded that such reductions (during the first two years of the crisis) reflect most probably supply-side problems: hospital budgets were cut by 40%, many clinics were understaffed due to the suspension of public hiring, shortages in medical supplies were encountered rather frequently whilst patients in many cases had to bribe medical staff in order to be given priority especially in overloaded hospitals with large queues. It has also been reported that although physician visits for issues related to chronic diseases have been largely met, this was accomplished with an increase in out-of-pocket expenditures and cuts in family budgets [21]. It is worth noting that the percentage of such illegal payments for health care services in Greece has been estimated to be more than 20% of the total private expenditure [6] whilst the out-of-pocket payments in Greece was one of the highest among OECD countries [2].

As it can be seen in Table 1, there was a tremendous cut in hospital expenses in the period 2009–2013. Specifically, total expenses in 2013 were cut by almost 52% since 2009. A closer look at the evolution of expenses over time reveals that the sharpest decline occurred between 2011 and 2012. In particular, almost 35 out of the 52 percentage points of the total cut took place in the transition from 2011 to 2012.

In the same period, as shown by Table 1, there was also a substantial reduction in the number of medical and other staff amounting to almost -10% and -15% respectively, reflecting the imposed suspension of recruitments in the public sector.

## Methods

### Efficiency assessment using Data Envelopment Analysis

Improving efficiency has become an increasingly important target for hospital managers. One of the most commonly used tools for efficiency measurement is Data Envelopment Analysis (DEA) [22]. DEA is applied to a set of homogenous units -the so-called decision making units, DMUs- and seeks to maximize each unit’s efficiency as it is defined through the ratio of weighted sum of outputs over weighted sum of inputs. The concept behind the method is that it allows each unit to weigh production inputs and outputs in a way so as to achieve the maximum possible efficiency compared to the other units in the sample. Put differently, each unit is allowed, in essence, to consider its own production practice as best by weighting inputs and outputs in the most preferable way [23].

In mathematical terms, the afore-mentioned objective in conjunction with certain assumptions about the producible production points (i.e. efficient input-output combinations permitted by the technology) leads to the formulation of a fractional programming problem. The solution of an equivalent linear program identifies a set of units that are deemed as efficient and all other units are deemed as inefficient. Determination of fully efficient units (also known as best practice units) enables the construction of a piece-wise linear frontier, the so-called “best practice frontier” which isolates potentially efficient units (all points on the frontier) from inefficient ones (all productively attainable points surrounded –enveloped- by the frontier). All units residing on the frontier can be thought of as either the ones that produce a certain level of output using the lowest allowed amount of inputs or the ones than produce the highest attainable level of output using a certain amount of inputs. Thus, all these technically efficient units on the frontier are assigned an efficiency score of 1 (100%) whereas technically inefficient ones are assigned a positive score less than 1 (less than 100%). The percentage score of an inefficient unit can be derived in one of two alternative ways referred to as input-orientated and output-orientated efficiency scores. In input-orientation the score represents the maximum allowed equiproportionate (i.e. radial) reduction of its inputs that is still capable of producing the same level of output. Accordingly, in output-orientation the score reflects the maximum equiproportionate (radial) expansion of its outputs that can be produced using the same level of inputs. In geometric terms this can be thought of as the maximum allowed contraction or required expansion of the unit’s input/output position ray until the unit has reached the efficient frontier.

It is evident that DEA is a non-parametric method which is based solely on the observed input-output combinations of the units in the sample without any assumptions concerning the form of the production function. The term Data Envelopment Analysis (DEA) was introduced by Charnes et al. [24] in their paper in 1978 which was based on the previous relevant research of Farell (1957) [25]. The method has been used in numerous sectors, including healthcare in which its first applications date back to the1980s [26–27]. An advantage of the method is its ability to handle multiple inputs as well as multiple outputs, without the requirement of a common denominator of reference.

Formally, given a set of *n* DMUs (*DMU*_*r*_, *r* = 1,2, …*p*, …*n*) each one consuming m inputs (*x*_1*r*_, *x*_2*r*_, …, *x*_*mr*_) to produce s outputs (*y*_1*r*_, *y*_2*r*_, …, *y*_*sr*_), the input oriented efficiency $\theta_{p}^{*}$ of unit p, is given by the solution of the following linear programming problem:

$$\theta_{p}^{*}=\text{max}\sum_{j=1}^{s}u_{jp}y_{jp}-w_{p},\;p\;\in \;\left\{1,2,\ldots ,n\right\}.$$

Subject to:

$$\sum_{i=1}^{m}v_{ip}x_{ip}=1\;.$$

$$\sum_{j=1}^{s}u_{jp}y_{jr}-w_{p}-\sum_{i=1}^{m}v_{ip}x_{ir}\leq 0\;\forall \;r=1,2,\ldots n.$$

$$u_{jp}\geq \varepsilon ,\;v_{ip}\geq \varepsilon \;\forall \;i,j.$$

$$w_{p}\in \mathbb{R}$$

Where *ε* is a non-Archimedean infinitesimal value for forestalling weights *u*_*jp*_, *v*_*ip*_ to be zeroed. The formulation stated above refers to the BCC model [28] which assumes variable returns to scale. Omitting *w*_*p*_ in the above formulation the linear program is converted to what is known as CCR model [24] which assumes constant returns to scale. When the BCC model is applied, the sign of *w*_*p*_ that comes out from the solution of the linear program identifies the nature of returns to scale.

### Window-DEA analysis

A drawback of DEA is that efficiency measures are defined relative to the best practice frontier of the sample under examination and consequently DMUs deemed as efficient are efficient only in relation to others in the particular sample [29]. Therefore, it is not meaningful in general to compare the scores between two different samples as all calculations are based on different best practice frontiers whose differences are not known. Consequently, even the efficiency comparison of the same set of units in two different time periods is questionable. In order to overcome this subtle point of DEA that hampers the comparison of efficiency scores over time (i.e. efficiency changes), one could move from the so-called contemporaneous perspective, where a unique frontier is derived for each time period, to the so-called intertemporal perspective where a single common frontier which spans the whole period is defined [30]. The basic idea within this latter framework is to regard each unit as if it were a different unit in each of the reporting periods. Thus, the performance of a unit in a particular period is compared with its own performance in other periods as well as with the performance of other units. Although, within this latter perspective, a year-to-year comparison can be carried out, one has to bear in mind that this approach implicitly assumes that there are no substantial technical changes over the entire time period. (i.e. the technological frontier is fixed). This assumption, however, cannot always be considered valid, especially when long time periods are analyzed, since production conditions may have substantially altered between distant years.

A compromise between contemporaneous and inter-temporal analyses is the so-called window analysis where DEA is applied successively on overlapping time periods of constant width (called a window). Once the window width has been specified all observations within it are viewed and examined in an inter-temporal manner referred to as locally inter-temporal analysis [31]. The method was initially proposed by Charnes et al. [32] in order to measure efficiency in cross sectional and time varying data. Furthermore, when window-DEA is applied, the number of observations taken into account is multiplied essentially by a factor equal to window’s width, which is quite useful when dealing with small sample sizes as it increases the discrimination capability of the method [33]. Therefore, two factors should be reconciled when choosing window width. The window should be wide enough to incorporate the minimum number of DMUs for the required discrimination but it should also be narrow enough to ensure that technological change within it is negligible and therefore it will not allow misleading or unfair comparisons between DMUs belonging to distant apart time periods [34].

A review of the literature shows that window analysis has been employed in DEA studies for a variety of purposes [30,35–41]. The method has also been applied in health care sector applications [34,42–44].

### The Malmquist productivity index

The assessment of productivity change over time together with its decomposition into efficiency changes and technology changes can be carried out using the so-called Malmquist index which was first introduced by Caves et al. [45] based on an idea of Malmquist [46]. Following Fare et al. [47] the input-based adjacent Malmquist index between time periods t and t+1 is given by

$$MPI\left(x^{t+1},y^{t+1},x^{t},y^{t}\right)={\left[\frac{d_{CRS}^{t}\left(x^{t+1},y^{t+1}\right)}{d_{CRS}^{t}\left(x^{t},y^{t}\right)}\;\frac{d_{CRS}^{t+1}\left(x^{t+1},y^{t+1}\right)}{d_{CRS}^{t+1}\left(x^{t},y^{t}\right)}\right]}^{1/2},$$

Where $d_{CRS}^{t}\left(x^{t},y^{t}\right)$ and $d_{CRS}^{t+1}\left(x^{t},y^{t}\right)$ represent the distance functions [48] of the production bundle (*x*_*t*_, *y*_*t*_) from the CRS technology frontiers in periods t and t+1 respectively whilst $d_{CRS}^{t}\left(x^{t+1},y^{t+1}\right)$ and $d_{CRS}^{t+1}\left(x^{t+1},y^{t+1}\right)$ represent the corresponding distance functions for the production bundle (*x*_*t*+1_, *y*_*t*+1_), i.e.

$$d_{CRS}^{\tau }\left(x^{\varphi },y^{\varphi }\right)=max\left\{\theta >0:\left(x^{\varphi }/\theta ,y^{\varphi }\right)\;\in \;T_{CRS}^{\tau }\right\},$$

where

$$T_{CRS}^{\tau }=\left\{\left(x^{\tau },y^{\tau }\right):\;\begin{array}{c} \sum_{j=1}^{n}\lambda_{j}^{\tau }x_{j,i}^{\tau }\leq x_{i}^{\tau }\;,\;i=1,2,\ldots m,\\ \sum_{j=1}^{n}\lambda_{j}^{\tau }y_{j,r}^{\tau }\geq y_{r}^{\tau }\;,r=1,2,\ldots ,s,\\ \;\lambda_{j}^{\tau }\geq 0,\;j=1,2,\ldots ,n \end{array}\right\},$$

for *τ* = {*t*, *t* +1} and *φ* = {*t*, *t* +1} and are equal to the reciprocal of the Farell measures of technical efficiency.

The *MPI* index defined above can equivalently be written as

$$MPI\left(x^{t+1},y^{t+1},x^{t},y^{t}\right)=\frac{d_{CRS}^{t+1}\left(x^{t+1},y^{t+1}\right)}{d_{CRS}^{t}\left(x^{t},y^{t}\right)}{\left[\frac{d_{CRS}^{t}\left(x^{t+1},y^{t+1}\right)}{d_{CRS}^{t+1}\left(x^{t+1},y^{t+1}\right)}\;\frac{d_{CRS}^{t}\left(x^{t},y^{t}\right)}{d_{CRS}^{t+1}\left(x^{t},y^{t}\right)}\right]}^{1/2},$$

The first term is equal to the Farell technical efficiency measure at period t divided by the Farell technical efficiency measure at period t+1 and therefore reflects the efficiency change component in productivity change. In other words, the term indicates whether the hospital has moved closer to the CRS-frontier (i.e. catching-up to the frontier). The second term is equal to the geometric mean of the shifts in the CRS technology observed at the production bundles (*x*_*t*_, *y*_*t*_) and *x*_*t*+1_, *y*_*t*+1_ respectively and therefore reflects the technological change component in productivity change (i.e. shift in the frontier).

The component of technical efficiency change can be further decomposed into pure technical efficiency change and scale efficiency change. Assuming the variable returns to scale technologies $T_{VRS}^{t}$ and $T_{VRS}^{t+1}$ for the two-time periods τ = {t, t+1}, i.e.

$$T_{VRS}^{\tau }=\left\{\left(x^{\tau },y^{\tau }\right):\;\begin{array}{c} \begin{array}{c} \sum_{j=1}^{n}\lambda_{j}^{\tau }x_{j,i}^{\tau }\leq x_{i}^{\tau }\;,\;i=1,2,\ldots m,\\ \sum_{j=1}^{n}\lambda_{j}^{\tau }y_{j,r}^{\tau }\geq y_{r}^{\tau }\;,r=1,2,\ldots ,s, \end{array}\\ \sum_{j=1}^{n}\lambda_{j}^{\tau }=1,\\ \lambda_{j}^{\tau }\geq 0,\;j=1,2,\ldots ,n \end{array}\right\},$$

and the corresponding distance functions

$$d_{VRS}^{\tau }\left(x^{\varphi },y^{\varphi }\right)=max\left\{\theta >0:\left(x^{\varphi }/\theta ,y^{\varphi }\right)\;\in \;T_{VRS}^{\tau }\right\},$$

where *τ* = {*t*, *t* +1} and *φ* = {*t*, *t* +1}

the *MPI* can equivalently be written as

$$MPI\left(x^{t+1},y^{t+1},x^{t},y^{t}\right)=\frac{d_{VRS}^{t+1}\left(x^{t+1},y^{t+1}\right)}{d_{VRS}^{t}\left(x^{t},y^{t}\right)}\frac{\frac{d_{CRS}^{t+1}\left(x^{t+1},y^{t+1}\right)}{d_{VRS}^{t+1}\left(x^{t+1},y^{t+1}\right)}}{\frac{d_{CRS}^{t}\left(x^{t},y^{t}\right)}{d_{VRS}^{t}\left(x^{t},y^{t}\right)}}{\left[\frac{d_{CRS}^{t}\left(x^{t+1},y^{t+1}\right)}{d_{CRS}^{t+1}\left(x^{t+1},y^{t+1}\right)}\;\frac{d_{CRS}^{t}\left(x^{t},y^{t}\right)}{d_{CRS}^{t+1}\left(x^{t},y^{t}\right)}\right]}^{1/2},$$

Thus, the Malmquist productivity index can be thought of as the product of three terms representing the changes attributed to pure technical efficiency, scale efficiency and technology. Taking the reciprocal of the indices calculated above [47], values greater than unity are meant to indicate progress whilst values smaller than unity indicate regress. It should be noted however that technological change can be calculated in a different way using the so-called base period Malmquist index introduced by Berg et al. [49].

A review of the literature in the health sector in Greece shows that the method has been employed for the assessment of productivity change in hospitals [50–51], hospital clinics [52–53] and dialysis facilities [54].

### Bias correction with bootstrapping

One of the disadvantages of DEA is that statistical inference is difficult to be applied due to the implied assumption of the method that the whole distance of a DMU from the efficient frontier reflects solely its inefficiency. In reality, this distance reflects inefficiency as well as sampling variability and noise because input and output data are normally subject to errors. Furthermore, given the assumption that noise does not exist, the estimated empirical technology can only be a subset of the true but unknown technology, and therefore DEA scores will be upwards biased [55]. In order to overcome this shortcoming of biased DEA scores due to sampling variability Simar and Wilson [56] proposed a methodology, which is based on bootstrap techniques [57] and allows determining the statistical properties of DEA estimators.

In the present study, their bootstrapping approach [58] was applied in order to calculate the bias-corrected Malmquist indices together with estimations of their confidence intervals. Thus, the method allows for examination whether increases or decreases in productivity are significant in a statistical sense. In other words, the method allows for the conclusion of whether a result indicates real progress/regress or is a coincidence due to sampling variation.

## Materials and methods

### Sample

The study examined the efficiency of a sample of 107 Greek NHS hospitals over the five-year period, 2009–2013. Twenty-four out of the 131 hospitals of the Greek NHS were excluded due to the idiosyncratic nature of health care services they provide [59–61]. More specifically, nine psychiatric, four anticancer/tumor, two dermatological, one maternity, one ophthalmological, one special diseases, one thoracic diseases, one pathological and four pediatric hospitals were excluded from the analysis in order to increase homogeneity of the remaining units. Going one step further towards increasing homogeneity and to permit their comparability, these 107 hospitals were additionally classified into four groups according to their size and the mixture/range of services they provide [59, 62–63]. Thus, under this classification the four groups A, B, C, D were assigned with N_A_ = 21, N_B_ = 33, N_C_ = 30 and N_D_ = 23 members respectively with corresponding sizes spanning within the ranges of <85, 85–190, 190–400, >400 beds. At this point, it should be noted that three hospitals could not be classified as belonging always to the same group throughout the whole five-year period of the study due to reductions/augmentations in their bed capacity. In order to maintain exactly the same members within each group, those three hospitals were considered to belong to the group into which they were assigned most of the time (i.e. 3 or 4 out of the 5 years).

All relevant data were collected from sources of Ministry of Health and Welfare. The data were given in (annual) summarized form and therefore no medical records or any other information concerning patients/caregivers/staff were used in the study. The study was also approved by the Ethical Committee of the Hellenic Open University

### Model specification

In DEA, the set of variables (inputs and outputs) that need to be included in the model should meet the following criteria: inputs should cover the full range of resources used; outputs should capture all activity levels and performance measures; furthermore, both, input and output variables, should constitute a set of factors common to all units under evaluation [33].

Thus, in accordance with published research [60,64–67] the actual variables selected for this study are among some of the most commonly used inputs and outputs affecting hospital efficiency. On the input side, labor and capital inputs were aggregated as follows: labor inputs were measured in terms of absolute numbers of staff (in full time equivalents- FTE) classified separately as physicians and other hospital employees. The separation was considered necessary since the former are fundamentally different as they enjoy the primary “decision rights” for patient care. Physicians are the most dominant and influential components in the entire production process, with little or no interference from management and it has been reported [68] that their decisions, directly or indirectly, may eventually account for as much as 80–90% of the total health care expenditure in any system. Capital input, as in most relevant studies [60,64–67], was proxied by the number of hospital beds assuming that invested capital per bed is similar throughout the hospitals in the sample. This assumption, in the present study, is expected to be valid considering the high degree of homogeneity of the four groups in the whole sample.

As regards outputs, patients’ health gain is the ultimate measure of output against which hospital activity should be assessed. Since practical difficulties limit this outcomes approach [69], output is usually measured as an array of health care services that supposedly improve patients’ health. Thus, in this study the multiplicity of hospital services was aggregated into three main outputs: inpatient cases, surgeries and outpatient visits. As far as inpatient services are concerned the number of cases was chosen rather than the (alternative choice of) number of inpatient days in order to avoid distortions imposed by variations in average length of stay due to higher/lower occupancy rates [43]. Furthermore, surgeries were counted separately as they constitute a fundamentally different part of inpatient services (compared to those of general medical) which usually account for a substantial volume of the total inpatient work. Thus, the total number of surgeries was included as a separate output variable in order to capture the workload of this special inpatient component. Finally, the number of outpatient visits was included as the third output variable representing the volume of outpatient services of the hospitals.

All DEA models in this study were formulated as input-oriented because of the public character of the hospitals which implies that hospital managers cannot seek for output increment but for reduced input usage instead [70].

Finally, a 2-year window width was chosen and therefore four overlapping windows were analyzed over the 5-year study period. The narrow window width was decided since it provides the minimum common ground that allows the year-to-year efficiency comparisons without the possible distortion that would have been imposed if a wider one had been chosen. At the same time, the 2-year width is considered sufficiently large -as far as the discrimination of the method when applied on small groups is concerned—since, it doubles the number of DMUs in each window. It is evident that the choice of a wider window could not be justified –especially within the years of the financial crisis of the country that are characterized by substantial changes in the health care sector affecting, among others, the applied technology. Thus, a wider window would most likely have led to unfair or non-realistic comparisons among hospitals in distant apart years.

## Empirical results

Descriptive statistics for input and output variables for the four groups of hospitals over the four 2-year windows are presented in Table 2.

**Table 2:** Summary statistics of input and output variables.

Window	Inputs			Outputs			Inputs			Outputs		
	I_1_	I_2_	I_3_	O_1_	O_2_	O_3_	I_1_	I_2_	I_3_	O_1_	O_2_	O_3_
**Mean**	**Group A: < 85 beds *(N = 21)***						**Group C: 190–400 beds (*N = 30)***					
2009–10	53.7	37.7	106.0	2,296.1	566.6	36,794.0	268.9	191.1	569.1	18,533.6	4,009.7	107,378.9
2010–11	53.9	35.3	104.5	2,370.3	589.4	35,537.0	267.5	188.1	557.2	19,406.4	3,889.5	101,438.7
2011–12	51.6	27.2	101.6	2,354.5	662.2	32,367.4	271.2	160.5	554.0	19,691.3	3,777.5	116,817.1
2012–13	48.5	26.4	98.7	2,223.7	660.1	30,288.5	272.4	157.9	544.3	19,536.8	3,868.4	114,394.2
**Stdev**												
2009–10	24.3	22.4	51.7	1,508.5	386.3	17,614.1	62.5	75.7	176.6	5,963.4	1,514.7	56,707.1
2010–11	24.1	19.3	48.9	1,521.0	438.3	15,819.3	62.2	74.4	160.8	6,143.2	1,580.6	53,722.3
2011–12	23.2	12.8	44.6	1,523.7	448.9	12,898.7	64.1	63.4	148.2	6,210.8	1,530.9	44,122.8
2012–13	23.4	12.2	44.6	1,453.6	404.4	11,087.0	69.9	67.5	145.2	5,945.5	1,420.0	39,966.4
**Median**												
2009–10	59.5	34.5	99.5	1,951.0	477.0	37,720.7	263.0	172.5	515.5	17,824.1	3,662.5	104,560.0
2010–11	59.0	33.0	98.0	2,101.0	482.5	34,042.5	262.5	171.0	533.5	18,778.2	3,565.5	98,839.5
2011–12	59.0	25.0	97.0	2,100.5	543.0	32,078.5	262.5	142.0	559.5	19,047.0	3,467.5	111,452.5
2012–13	49.5	25.0	98.0	1,975.0	573.5	30,083.0	261.5	135.0	554.5	19,649.5	3,650.0	110,190.0
**Min**												
2009–10	18.0	8.0	32.0	388.0	42.0	3,940.0	161.0	73.0	263.0	8,020.0	1,575.0	13,612.0
2010–11	19.0	8.0	41.0	334.0	44.0	8,022.0	177.0	73.0	261.0	8,020.0	1,054.0	13,612.0
2011–12	20.0	8.0	41.0	60.0	44.0	8,022.0	177.0	73.0	261.0	10,836.0	1,054.0	43,066.0
2012–13	10.0	8.0	32.0	60.0	115.0	8,601.0	153.0	61.0	241.0	9,644.0	1,309.0	37,486.0
**Max**												
2009–10	92.0	110.0	214.0	5,548.0	1,597.0	71,419.0	392.0	375.0	989.0	31,820.0	8,157.0	249,208.0
2010–11	92.0	100.0	214.0	5,552.0	1,898.0	71,301.0	394.0	375.0	968.0	34,559.0	7,856.0	232,887.0
2011–12	92.0	52.0	182.0	5,552.0	1,898.0	63,480.0	422.0	333.0	828.0	34,559.0	7,856.0	229,254.0
2012–13	81.0	52.0	182.0	5,128.0	1,720.0	55,121.0	422.0	333.0	828.0	33,340.0	7,448.0	206,441.0
**Mean**	**Group B: 85–190 beds (*N = 33)***						**Group D: >400 beds (*N = 23)***					
2009–10	125.3	94.0	261.7	7,710.7	2,120.6	65,724.6	613.5	490.0	1,130.1	39,919.9	8,741.0	194,266.4
2010–11	127.5	91.4	257.4	8,158.0	2,118.5	64,307.3	615.2	446.1	1,084.1	44,214.9	8,694.2	178,577.0
2011–12	126.5	74.5	256.1	8,010.7	2,074.4	64,350.4	615.2	390.7	1,040.2	45,978.4	8,875.3	167,533.7
2012–13	126.3	72.3	248.6	7,667.7	2,008.2	63,469.9	613.7	363.7	1,030.7	45,951.9	8,851.0	167,304.4
**Stdev**												
2009–10	31.8	40.7	78.0	2,917.3	1,409.4	26,267.9	153.4	113.2	275.3	13,532.9	3,739.0	80,728.0
2010–11	31.3	40.3	77.7	2,995.7	1,274.1	23,317.1	155.8	113.5	255.3	14,364.4	3,770.6	70,072.0
2011–12	29.9	28.7	80.8	2,951.0	1,116.1	23,022.5	155.5	98.5	222.2	14,679.7	4,095.4	57,741.1
2012–13	31.0	28.5	77.5	3,065.8	1,071.7	25,740.6	148.6	108.1	229.0	14,362.6	4,113.1	55,730.0
**Median**												
2009–10	119.0	86.0	237.0	6,956.5	1,691.5	59,177.0	616.5	483.0	1,089.0	40,421.5	8,085.0	171,414.5
2010–11	120.0	84.5	238.0	7,374.0	1,786.5	58,567.5	606.0	430.5	1,057.0	44,885.0	7,955.0	168,672.5
2011–12	120.0	63.0	243.0	7,374.0	1,830.0	58,112.5	599.5	369.0	1,022.0	48,244.5	7,955.0	164,438.0
2012–13	120.0	63.0	233.0	6,954.5	1,818.5	58,112.5	617.5	347.0	1,027.5	46,831.0	8,185.0	163,625.0
**Min**												
2009–10	71.0	39.0	137.0	2,975.0	514.0	20,601.0	396.0	238.0	681.0	19,429.0	2,062.0	76,736.4
2010–11	83.0	35.0	125.0	3,253.0	602.0	23,747.0	396.0	215.0	661.0	21,974.0	2,048.0	40,107.0
2011–12	83.0	35.0	125.0	3,253.0	518.0	31,965.0	414.0	215.0	661.0	21,075.0	1,892.0	40,107.0
2012–13	80.0	29.0	116.0	2,942.0	518.0	15,506.0	405.0	118.0	661.0	21,075.0	1,873.0	79,003.0
**Max**												
2009–10	200.0	225.0	467.0	15,046.0	7,046.0	141,055.0	949.0	831.0	1,764.0	69,030.0	19,529.0	422,688.0
2010–11	200.0	225.0	529.0	15,173.0	7,044.0	140,348.0	949.0	706.0	1,764.0	68,816.0	18,883.0	399,779.0
2011–12	200.0	157.0	529.0	15,173.0	6,055.0	141,320.0	933.0	706.0	1,548.0	68,816.0	18,913.0	309,884.0
2012–13	208.0	157.0	529.0	13,807.0	5,998.0	151,516.0	948.0	706.0	1,671.0	66,417.0	18,913.0	292,422.0

I1 = Beds.

I2 = Physicians.

I3 = Other staff

O1 = Inpatient cases.

O2 = Operations.

O3 = Outpatient visits

Average year-specific and window-specific VRS efficiency scores are presented in Table 3 which has been constructed in the following way [71]. Columns represent years and rows represent windows. Thus, the intersection of a row with a column represents a value in the context of a year within a specific window. It is clear that a hospital can have different efficiency scores for the same year in the context of different windows. More specifically, for a 2-year width window, each year will participate twice in two adjacent windows (excluding the first and last years of the study period which participate only once). Consequently, when reading the table vertically (following the so-called column view) one can see possible efficiency alterations for a hospital in the same year measured against the efficient frontiers of the two different windows it participates. Thus, any efficiency difference, in essence, reflects the impact on the efficient frontier due to changing half of the units (by adding a later year and removing the earliest one from the window) and therefore vertical fluctuation provides an indication for the stability of efficiency results for each year across the two different windows it participates. According to Cooper et al. [23], a hospital that is efficient in one year regardless of the window is said to be stable in its efficiency rating.

**Table 3:** Mean VRS and scale efficiencies.

*Window*	*Group A (N = 21)*					*entire window (N = 42)*	*Group B (N = 33)*					*entire window (N = 66)*	*Group C (N = 30)*					*entire window (N = 60)*	*Group D (N = 23)*					*entire window (N = 46)*
	2009	2010	2011	2012	2013		2009	2010	2011	2012	2013		2009	2010	2011	2012	2013		2009	2010	2011	2012	2013	
**Mean VRS efficiency**																								
2009–10	85.5	87.0				86.2	85.6	85.5				85.5	88.5	85.9				87.2	89.6	90.7				90.2
2010–11		87.1	91.1			89.1		89.4	91.6			90.5		86.4	92.6			89.5		88.2	93.3			90.8
2011–12			91.4	89.0		90.2			90.6	90.3		90.5			91.9	88.2		90.1			93.9	91.6		92.7
2012–13				90.3	88.9	89.6				89.8	89.8	89.8				87.3	90.2	88.8				89.7	91.8	90.8
Common Year	85.5	87.1	91.2	89.6	88.9		85.6	87.4	91.1	90.1	89.8		88.5	86.1	92.2	87.8	90.2		89.6	89.5	93.6	90.7	91.8	
**St.Dev. (VRS)**																								
2009–10	15.9	16.5				16.1	12.2	10.8				11.4	12.9	12.2				12.5	11.9	12.5				12.1
2010–11		15.7	10.3			13.2		10.5	9.3			9.9		11.5	10.9			11.6		13.5	10.8			12.4
2011–12			9.9	11.4		10.6			9.3	9.2		9.2			10.6	12.4		11.6			10.6	10.9		10.7
2012–13				12.9	14.5	13.6				9.7	9.7	9.6				13.7	13.0	13.4				12.4	11.6	11.9
Common Year	15.9	15.9	9.9	12.0	14.5		12.2	10.8	9.2	9.4	9.7		12.9	11.8	10.7	13.0	13.0		11.9	12.9	10.6	11.6	11.6	
**Number of VRS efficient units**																								
2009–10	8	10				18	8	5				13	12	5				17	7	8				15
2010–11		10	9			19		7	14			21		8	16			24		7	15			22
2011–12			9	7		16			11	8		19			13	6		19			14	7		21
2012–13				8	11	19				7	9	16				8	12	20				8	9	17
**Mean scale efficiency**																								
2009–10	90.0	89.6				89.8	85.9	86.0				85.9	93.6	92.1				92.8	87.4	87.2				87.3
2010–11		89.3	92.6			91.0		89.1	93.0			91.0		93.6	94.6			94.1		86.4	90.8			88.6
2011–12			91.8	91.7		91.7			93.6	91.5		92.6			92.7	92.7		92.7			90.5	89.2		89.8
2012–13				93.2	94.2	93.7				92.0	89.7	90.9				93.1	94.1	93.6				90.2	92.0	91.1
Common Year	90.0	89.5	92.2	92.5	94.2		85.9	87.5	93.3	91.8	89.7		93.6	92.8	93.6	92.9	94.1		87.4	86.8	90.6	89.7	92.0	
**St.Dev. (scale)**																								
2009–10	16.4	15.3				15.7	16.3	14.5				15.3	8.8	9.7				9.2	10.7	11.9				11.2
2010–11		14.9	11.9			13.4		12.2	7.8			10.4		10.5	8.2			9.4		12.6	11.3			12.0
2011–12			12.0	10.2		11.0			7.8	10.8		9.4			8.1	7.9		7.9			11.2	13.7		12.4
2012–13				8.5	7.7	8.0				9.6	12.0	10.9				7.1	7.8	7.4				13.6	11.6	12.5
Common Year	16.4	14.9	11.8	9.3	7.7		16.3	13.4	7.8	10.2	12.0		8.8	10.1	8.1	7.4	7.8		10.7	12.1	11.1	13.5	11.6	
**Number of scale efficient units**																								
2009–10	5	6				11	6	3				9	7	2				9	3	4				7
2010–11		5	6			11		4	7			11		4	10			14		2	6			8
2011–12			4	2		6			8	6		14			7	4		11			6	3		9
2012–13				5	6	11				7	6	13				4	6	10				6	5	11
**Number of IRS-DRS units**																								
2009–10	10–6	9–6				19–12	22–5	23–7				45–12	14–9	15–13				29–22	19–1	17–2				36–3
2010–11		8–8	8–7			16–15		25–4	18–8			43–12		17–9	16–4			33–13		20–1	16–1			36–2
2011–12			8–9	9–10		17–19			16–9	20–7		36–16			17–6	20–6		37–12			15–2	18–2		33–4
2012–13				7–9	7–8	14–17				20–6	20–7	40–13				18–8	17–7	35–15				15–2	16–2	31–4

On the other hand, when reading the table horizontally one can see how the efficiency of a hospital changes from one year to another within the same window. Thus, these row views, in essence, make it possible to determine efficiency trends (i.e. whether a hospital exhibits improving, steady or deteriorating efficiency in the second year of a window against to the corresponding one in the first year). As noted in [72] the observation of “stability” and “trend” in window analysis reflects simultaneously both the absolute performance of a hospital over time and the relative performance of that hospital in comparison to the others in the sample. Average values of row and column values, for each section, are presented in the bottom row (labeled “common year”) and in the rightmost column (labeled “entire window”) respectively.

The results in Table 3, for average year-specific and window-specific efficiency scores suggest that there is considerable room for improvement. More specifically, the lowest and highest year-specific mean efficiency scores are [85.5%-91.4%], [85.5%-91.6%], [85.9%-92.6%] and [88.2%-93.9%] for groups A, B, C and D respectively. The detailed results (not presented here) showed that the hospitals that exhibit the lowest efficiency scores for each group are A1 (49.8%, year 2013, window 2012–13), B14 (57.7%, 2010, window 2009–10), C2 (55.2%, 2009, 2009–10) and D9 (57.6%, 2010, window 2010–11). In the same table, the number of fully VRS efficient hospitals is also presented. Thus, year 2011 within window 2010–11 accommodates the highest number of fully efficient units for groups B, C and D. More precisely there are 14, 16 and 15 fully efficient units accounting for 42.4%, 53.3% and 65.2% of the total number of units for groups B, C and D respectively. In group A, the highest number (11) of fully efficient units appears in year 2013 within windows 2012–13. In addition, the VRS efficiency scores for groups A and B show the highest and lowest spread respectively, as evidenced by their corresponding standard deviations (and minimum values not presented in the table). As it can also be seen from the Table 3, the window-specific efficiency scores appear to have an increasing trend until window 2011–12 after which they are reversed and start to deteriorate.

Efficiency trends within windows, on individual hospital basis, are shown in Table 4 which has been constructed in the following way: There are four columns per group corresponding to the four windows. Three symbols “**↗**”, “**↘**” and “**↔**”, are used to denote improvement, deterioration or steadiness of a hospital’s efficiency respectively, when going from the first to the second year within the same window. In addition, the symbol “**+**” is used in conjunction with these three symbols to denote a fully efficient unit in the implied year on the left or right hand side of the arrow. It is clear that only the three combinations “**↗+**”, “**+↘**”, and “**+↔+**” may arise. The first one, “**↗+**”, will be used when efficiency improvement starts from a non-efficient unit and leads to a fully efficient unit. Symmetrically, the second one, “**+↘**”, will be used when efficiency deterioration starts from a fully efficient unit and leads to a non-efficient unit. Finally, the third one, “**+↔+**”, will be used to denote the transition from a fully efficient unit to an also fully efficient unit. An extra summary column to the right of the four window-specific columns (labeled “inter-temporal trend”) is used in order to designate a constant inter-temporal (all-windows) behavior of a unit whenever there is one. Thus, if the same trend symbol appears for a hospital in all four windows the corresponding symbol **(↗**, **↘**, or **↔)** is placed in this summary column. In addition, the extra symbol “**√**” is placed in this column whenever a hospital manages to be fully efficient in at least one year within every window. Therefore, it is evident that the combined presence of the “**√**” together with the symbol **↔** (i.e. **↔√**) indicates a fully efficient hospital throughout all years within all windows. Similarly, the presence of symbol “**√**” either sole or accompanying the symbols **↗** and **↘**, indicates an (inter-temporal) almost fully efficient unit which we shall call a “semi-efficient” unit. Finally, at the bottom of the table, a summary of column totals shows the number of occurrences of each trend symbol. It is worth noting that the total number of occurrences of the “**+**” symbol on the left (or right) side of a trend arrow coincides with the total number of fully efficient hospitals in the year implied on the corresponding side of the arrow. For example, as can be seen in summary section of group B in Table 4, the symbol “+” appears 14 times on the right of trend arrows (9 x “**↗+**” and 5 x “**+↔+**”) in window (column) 2010–11. There are, therefore, 14 VRS fully efficient hospitals (within window 2010–11) in the year which is implied on the right of the particular trend arrow which, in this case, is 2011. Similarly, there are 7 (5 x “**+↔+**” and 2 x “**+↘**”) fully efficient hospitals in year 2010 within window 2010–11. Furthermore, it is evident that 5 hospitals (5 x “**+↔+**”) managed to be fully efficient in both years within window 2010–11.

**Table 4:** Efficiency trend (VRS).

Group A: <85 beds						Group B: 85 < Beds < 190						Group C: 190 < Beds < 400						Group D: >400 beds					
*ID*	*Window*				*Inter-temporal trend*	*ID*	*Window*				*Inter-temporal trend*	*ID*	*Window*				*Inter-temporal trend*	*ID*	Window				*Inter-temporal trend*
	2009–10	2010–11	2011–12	2012–13			2009–10	2010–11	2011–12	2012–13			2009–10	2010–11	2011–12	2012–13			2009–10	2010–11	2011–12	2012–13	
A1	↗	↗	↘	↘	∙	B1	↗	↗+	+↘	+↘	∙	C1	+↘	↘	↘	↗	∙	D1	↗	↗+	+↘	+↘	∙
A2	↗	↗	↗	↘	∙	B2	↗	↗	↗	↘	∙	C2	↗	↘	↘	↗	∙	D2	+↔+	+↔+	+↔+	↗+	√
A3	+↔+	+↔+	+↔+	+↘	√	B3	↗	↗	↘	↗	∙	C3	+↘	+↔+	+↘	↗	∙	D3	↘	↗	↘	↘	∙
A4	↗	↗	↗	+↔+	∙	B4	↘	↗	↗	↘	∙	C4	+↘	↗+	+↔+	+↔+	√	D4	↗+	+↔+	+↔+	+↔+	√
A5	↘	↗	↘	↘	∙	B5	↗	↗	↘	↘	∙	C5	+↔+	+↔+	+↘	+↘	√	D5	+↔+	+↔+	+↔+	+↔+	↔ √
A6	+↔+	+↔+	+↔+	+↔+	↔ √	B6	↘	↗	↔	↗	∙	C6	↘	↗	↗	↘	∙	D6	↗+	+↔+	+↘	↗+	√
A7	↗	↗	↘	↘	∙	B7	↗	↗+	↘	↘	∙	C7	↗	↗	↘	↗+	∙	D7	+↘	↗+	+↘	↗	∙
A8	↗+	↘	↘	↘	∙	B8	↗	↗+	+↘	↗+	∙	C8	↘	↗	↗+	+↔+	∙	D8	↗+	↗+	↘	↘	∙
A9	+↔+	+↘	+↘	↗+	√	B9	+↔+	+↔+	↘	↗	∙	C9	↗	↗+	+↘	↗	∙	D9	↘	↗	↗	↗	∙
A10	+↘	↘	↗	↗+	∙	B10	↗+	+↔+	+↘	↘	∙	C10	+↘	↗+	+↔+	↗+	√	D10	↘	↗	↘	↗	∙
A11	↘	↗	↗	↘	∙	B11	↗	↘	↘	↗	∙	C11	↘	↗	↗	↗	∙	D11	↘	↗+	+↘	↗+	∙
A12	↗+	+↔+	↗+	+↔+	√	B12	↘	↗+	+↔+	+↔+	∙	C12	+↔+	+↔+	+↔+	+↔+	↔ √	D12	↗+	↗+	↗+	+↘	√
A13	↗+	+↔+	+↘	↗+	√	B13	+↘	+↘	↗	↗	∙	C13	↘	↗	↗	↗	∙	D13	+↔+	+↔+	+↔+	+↔+	↔ √
A14	↘	↗+	+↘	↘	∙	B14	↘	↗	↗	↗	∙	C14	↘	↗	↗	↗	∙	D14	↗	↗	↘	↗+	∙
A15	+↔+	+↔+	+↔+	+↔+	↔ √	B15	↘	↗	↘	↗	∙	C15	↗	↗	↗	↗+	↗	D15	↗	↗	+↘	↗	∙
A16	+↔+	+↔+	+↘	+↔+	√	B16	↘	↗	↗	↘	∙	C16	+↔+	↗+	+↔+	+↔+	√	D16	↘	↗	↘	↗	∙
A17	↘	↗	↗	↘	∙	B17	+↘	+↔+	+↔+	↗+	√	C17	↘	↗	↔	↗+	∙	D17	↗	↗+	+↘	↗	∙
A18	+↘	+↘	↗+	↗+	√	B18	↗	↗	↘	↗	∙	C18	↗	↗+	+↘	↘	∙	D18	↗+	+↔+	+↘	+↔+	√
A19	↗+	+↘	↗+	+↔+	√	B19	↗	↗	↘	↗+	∙	C19	+↘	↗+	↘	↗	∙	D19	+↘	↗+	+↘	↗	∙
A20	+↘	↗+	+↔+	↗+	√	B20	↘	↗+	↗+	+↘	∙	C20	↘	↗+	↗	↘	∙	D20	↗	↘	↘	↗	∙
A21	↗+	+↔+	+↘	+↘	√	B21	+↘	↗+	+↔+	↗+	√	C21	↗	↗+	+↘	+↘	∙	D21	+↘	↗+	+↔+	+↔+	√
						B22	↘	+↔+	+↔+	+↘	∙	C22	↘	↗	↗	↘	∙	D22	+↘	+↔+	+↔+	+↔+	√
						B23	↗	↗	↗	↗+	↗	C23	↘	↗	↘	↗	∙	D23	↗	↗	↗	↗	↗
						B24	+↘	↗	↗+	+↔+	∙	C24	↘	↗	↘	↗+	∙						
						B25	↗	↗+	+↘	↗	∙	C25	↘	↗	↘	↗+	∙						
						B26	↘	↗	↔	↗	∙	C26	+↔+	+↔+	+↔+	+↔+	↔ √						
						B27	+↔+	+↘	↘	↘	∙	C27	+↔+	+↔+	+↘	↘	∙						
						B28	+↔+	+↔+	+↔+	+↔+	↔ √	C28	+↘	+↔+	+↘	↗	∙						
						B29	↗	↘	↗	↘	∙	C29	+↘	+↔+	+↘	+↔+	√						
						B30	↘	↗+	+↘	↘	∙	C30	↗	+↔+	↘	↗	∙						
						B31	↗	↘	↗	↗	∙												
						B32	↗	↘	↗	↗	∙												
						B33	+↔+	↗+	+↔+	+↔+	√												
Column totals—————————					Sum (%) of totals	Column totals—————————					Sum (%) of totals	Column totals—————————					Sum (%) of totals	Column totals—————————					Sum (%) of totals
↗	4	7	5	0	16 (19.0)	↗	14	13	9	12	48 (36.4)	↗	7	12	7	11	37 (30.8)	↗	6	7	2	9	24 (26.1)
↗+	5	2	3	5	15 (17.9)	↗+	1	9	2	5	17 (12.9)	↗+	0	8	1	6	15 (12.5)	↗+	5	8	1	4	18 (19.6)
+↔+	5	7	4	6	22 (26.2)	+↔+	4	5	6	4	19 (14.4)	+↔+	5	8	5	6	24 (20.0)	+↔+	3	7	6	6	22 (23.9)
+↘	3	3	5	2	13 (15.5)	+↘	4	2	5	3	14 (10.6)	+↘	7	0	8	2	17 (14.2)	+↘	4	0	8	2	14 (15.2)
↘	4	2	4	8	18 (21.4)	↘	10	4	9	9	32 (24.2)	↘	11	2	8	5	26 (21.7)	↘	5	1	6	2	14 (15.2)
↔	0	0	0	0	0 (0.0)	↔	0	0	2	0	2 (1.5)	↔	0	0	1	0	1 (0.8)	↔	0	0	0	0	0 (0.0)

Some interesting points emerge from a careful examination of Table 4. In terms of inter-window efficient units, as can be seen from the contents of the summary column for each group, the following apply: There are nine semi-efficient and two inter-efficient hospitals in group A. In group B, there is only one inter-efficient hospital and three semi-efficient ones. This is also the group with the lowest number of inter-temporal efficient units (i.e. semi-efficient plus inter-efficient). Group C has five semi-efficient hospitals and two inter-efficient ones while in group D there are seven semi-efficient and two inter-efficient units. In terms of inter-window trend (i.e. constantly upward/downward units) there are only three hospitals that appear to be trending always upward. These are hospitals B23, C15 and D23. It should be noticed also that the two smaller groups (A and D) are the ones with the highest number of inter-window efficient units.

**Fig 1:**
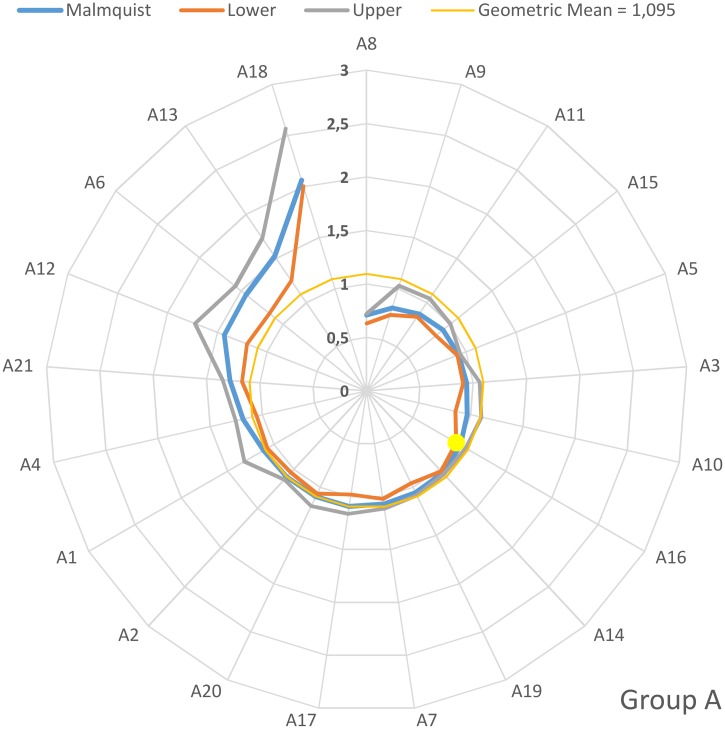
Malmquist productivity change (group A).

Table 3 summarizes also the results of average year-specific and window-specific scale efficiency. As can be seen, year-specific scale scores are relatively high and lie within the ranges [89.3–94.2], [85.9–93.6], [92.1–94.6] and [86.4–92.0] for groups A, B, C and D respectively. The diagrams in the second column of Fig. 1 depict graphically the average scale efficiency trend form year-to-year. It is evident that there is a downward dominating tendency for all groups in window 2009–10 (all groups deteriorate slightly, with the exception of group B that shows an inappreciable increment). In the following window, 2010–11, all groups appear to have a sharp upward trend which is followed by a more or less downward trend in window 2011–2012. More precisely one can see that groups B and D in window 2011–12 show a relatively sharp deterioration whilst groups A and C seem to remain almost constant.

Finally, in the last window 2012–13, although there is no clear common trend for all groups it is evident that there is an upward tendency since, with the exception of group B, all other groups show a clear improvement in their average scale efficiency scores when going from year 2012 to 2013. The sixth section of Table 3 presents the number of scale efficient hospitals (CRS). As can be seen the maximum number of scale efficient hospitals is achieved (on average) in the two instances of year 2011 for all groups (with the slight exception of group A). Finally, as far as scale inefficiency is concerned, one should be able to distinguish whether it is attributed to increasing or decreasing returns to scale. The last section in Table 3 summarizes the detailed findings concerning the nature of scale returns for each hospital. The contents of this section show that, with the exception of group A, for which relevant findings are almost equally divided, there is a clear dominance of hospitals operating at increasing returns to scale (IRS).

Table 5 presents the (geometric) average change of the estimations of the input-based Malmquist productivity index together with its decomposition into the components of efficiency change “*Eff*” (i.e. catching- up to the frontier) and technical change “*Tech*” (i.e. shift in the frontier). The index of the efficiency change component “*Eff*” is further decomposed into its two constituting indices of pure technical efficiency change “*Pure*.*eff*” and scale efficiency change “*Scale*”. The results are presented along with their 95% confidence intervals that were calculated applying the bias-corrected and accelerated (*BCα*) bootstrap methodology introduced by Efron and Tibshirani [57]. Since Malmquist indices are given in geometric rather than arithmetic means, the methodology was applied in the way described by Atkinson and Wilson [73] where the bootstrap procedure is initially used to calculate the confidence intervals for the arithmetic mean of the *log(index)* values and subsequently take their exponential values in order to derive the confidence intervals for the geometric mean.

**Table 5:** Decomposition of Malmquist productivity index (2009/2013).

Index	Geometric mean	95% condidence interval			Geometric mean	95% condidence interval		
		Lower	Upper			Lower	Upper	
	Group A				Group B			
MI	1.095	1.019	1.198	*	0.993	0.860	1,118	
Eff	1.118	1.048	1.220	*	1.118	1.045	1,191	*
Tech	0,979	0.922	1.067		0.889	0.857	0.920	*
Pure.Eff	1,059	0.963	1.186		1.055	1.015	1.101	*
Scale	1,056	0.961	1.259		1.059	1.003	1.122	*
	Group C				Group D			
MI	1.071	1.000	1.140	*	1.268	1.150	1,390	*
Eff	1.047	0.964	1.142		1.055	0.907	1,166	
Tech	1,024	0.975	1.083		1.202	1.138	1.294	*
Pure.Eff	1,031	0.947	1.102		1.011	0.960	1.076	
Scale	1,015	0.940	1.075		1.043	0.919	1.125	

MI: Malmquist productivity index

Pure.Eff: Pure technical efficiency change

Eff: Technical efficiency change

Scale: Scale efficiency change

Tech: Technological change

* Statistically significant

As can be seen from the empirical results of the Table 5, all groups of hospitals achieved statistically significant (at the 5% level) increases in productivity between 2009 and 2013 with the exception of group B that is shown to exhibit a (non-statistically significant) slight decrease. It is interesting to note that the larger hospital set (group D) is shown to exhibit the greater (statistically significant) productivity change by 26.8%. However, when the total productivity change is decomposed into efficiency and technical change, it appears clearly that the growth in productivity is primarily attributed (20.2%) to a progressive shift of the efficient frontier and secondarily (5.5%) to efficiency improvement. The results indicate that the dominance of the technology versus efficiency change is reversed for the other groups of hospitals. Specifically, in groups A and B, the contribution of efficiency change in productivity is positive by 11.8% whilst the corresponding contribution of the technology change component is negative by 2.1% and 11.1% respectively. Similarly, the statistically significant productivity improvement by 7.1% in group C is mainly due to efficiency improvement (4.7%) rather than to technology progress (2.4%).

Summing up, the results in Table 5 reveal that all indices have a positive contribution (>1) the only exception being the technology change index in groups A and B where it appears to participate negatively. However, only in group B does a negative contribution lead to a marginal (non-significant) productivity decline by 0.7%.

**Fig 2:**
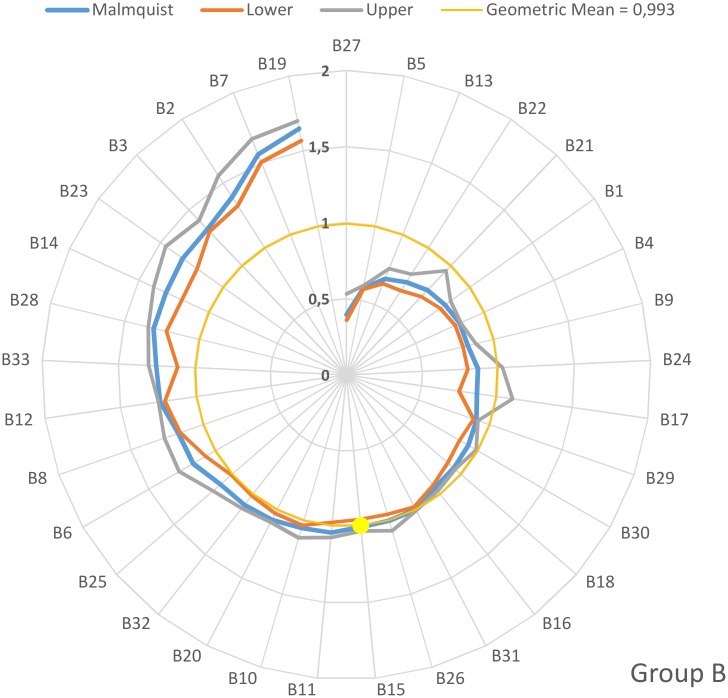
Malmquist productivity change (group B).

**Fig 3:**
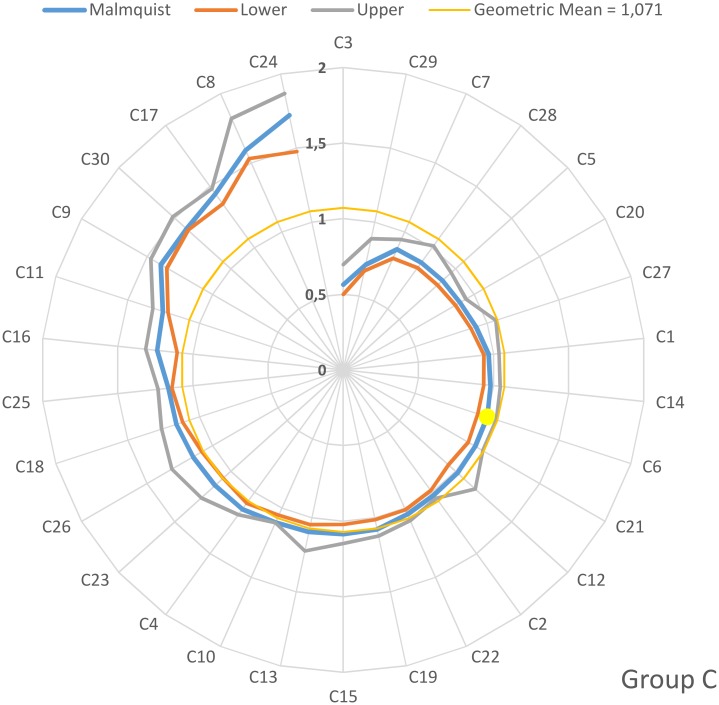
Malmquist productivity change (group C).

**Fig 4:**
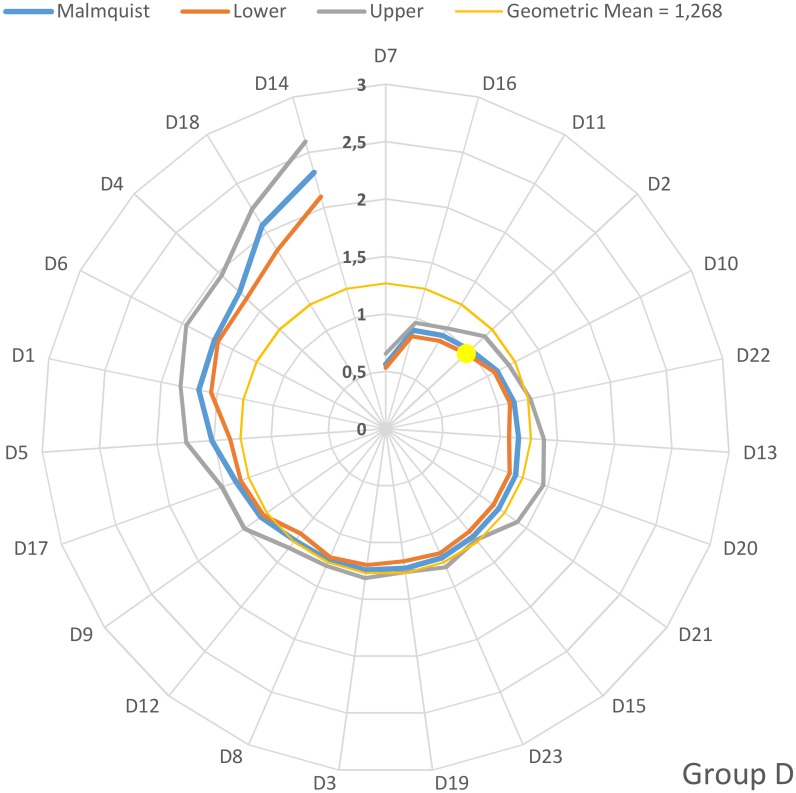
Malmquist productivity change (group D).

On an individual basis, the values of the Malmquist productivity indices for groups A, B, C and D are depicted in the four diagrams depicted in Figs 1–4. The hospitals have been arranged clock-wisely in ascending order according to their MPI values (blue line) whilst the first occurrence of an MPI value >1 is denoted by the yellow dots. The MPI line is surrounded by two spirals that correspond to the bootstrap calculated for upper and lower values for the 95% confidence interval. Furthermore, the geometric MPI mean for each group is depicted as a yellow circle. From the diagrams, it is easily deducted that the number of hospitals that experienced overall productivity progress (MPI>1) for the groups A, B, C and D are 14/21 (= 66.6%), 17/33 (= 51.5%), 21/30 (= 70%) and 20/23 (= 87.0%) respectively. The MPI values span in the range from 0.7–2.06 (group A), 0.39–1.64 (group B), 0.56–2.72 (group C) and 0.56–2.32 (group D).

## Discussion

The main objective of this study was to apply DEA to measure the efficiency of the Greek NHS hospitals during the period 2009–2013. The hospitals were categorized and allocated into four separate groups with common characteristics in order to increase their homogeneity. The DEA-Windows method was chosen for the assessment of their efficiency since (a) it leads to increased discrimination on the results especially when applied on small samples and (b) provides a means for the year-to-year comparison of the results. At the same time, the number of DMUs is doubled since, in essence, every actual hospital participates twice in each window and therefore the discrimination of the method is improved.

The findings showed that all four groups of hospitals were operating at relatively high levels of technical and scale efficiency over the whole period. In general, mean pure technical efficiency (VRS) for all groups was found to span approximately a range of values from 85.5% up to almost 94%. Mean scale efficiency was also found to lie within the range 85.9%-94.6%.

The analysis also concluded that there are only seven hospitals that can be considered all-windows best performers. Two of them belong to group A (A6, A15), one (B28) is a member of group B and two (C12, C26) are members of group C and finally two (D5, D13) belong to group D. These seven hospitals are the ones that managed to maintain their full pure technical efficiency in all eight instances of their evaluation against the four different window frontiers. This finding, therefore, suggests that hospital managers and policy makers should pay attention and examine closely and thoroughly their applied practices and technology profile since they can serve as benchmarks for the others. In addition, apart from this small number of the seven all-window best performers, the DEA-window analysis identified also a respectable number of all-window very good performers which we called “semi-efficient”. These are the hospitals that managed to remain fully efficient at least once in every window. It is worth noting that when their results were scrutinized on an individual hospital basis it was found that almost all of them managed to maintain a very high VRS efficiency score in the years that they were not fully efficient. On the other end of the spectrum, the analysis also reported 33 hospitals as being consistently inefficient over all windows. These are 33 hospitals that never managed to achieve a pure technical efficiency score of 100% in any of the years and they are distributed as 6, 14, 7 and 6 in the four groups respectively. Although not all of them can be considered as all-windows worst performers the worst ones are indeed among them. From a managerial point of view, it is clear that this group of hospitals needs also to be scrutinized closely in order to identify peculiarities and possible sources of inefficiency. Hospitals A2, B14, C2 and D9 are the ones with the lowest average efficiency scores (65.8%, 73.3%, 57.7%, 62.9% respectively) over all windows. Among the factors that may explain this finding is the fact that all output/input ratios of these hospitals are among the lowest of their groups throughout all years. Furthermore, hospital A2 is shown to have the lowest occupancy rate (29%) whilst hospitals C2 and D9 exhibit almost a twofold ALoS compared to the average values of their groups. Hospital A2 is located on a rather small island and therefore the low occupancy rate is expected due to the low population. On the other hand, hospitals C2 and D9 are big hospitals located in Athens and are among those covering the more difficult and complicated cases that require longer stays.

As far as efficiency trend is concerned, the finding that emerged from window analysis is the following. Although the results do not reveal the existence of a clear and consistent inter-temporal trend in any of the four groups there is, however, an indication that year 2011 constitutes a turning point in the whole 5-year period since the upward efficiency trends for all groups in window 2010–11 are turning downwards in window 2011–12. A similar finding is documented in another study [61] according to which the middle-sized hospitals (100–400 beds) of the Greek NHS exhibit efficiency improvement in the period 2009–2011.

Empirical results also showed that the hospitals under study were operating at respectable levels of scale efficiency from 85.9% up to 94.6%. Detailed results, summarized in Table 3, show that a small number of hospitals were operating at optimal size (characterized by constant returns to scale), though many others were operating close to their optimal size. Furthermore, by examining individual scale efficiency findings, it was concluded that the hospitals under study exhibit a mix of decreasing and increasing returns to scale at current levels of output. Interestingly, the pattern of scale inefficiency indicates that most of the hospitals are operating in an area of increasing returns to scale implying that they could benefit from increasing the scale of their operations. At this point two things need also to be taken into account. First, the size of each hospital should be interpreted relatively in the context of the group it belongs to and therefore it should not be seen as an absolute “big” or “small” number. Second, scale inefficiency for many hospitals account for as little as 5% or even less. This implies that the potential for their improvement through resizing is rather limited. These findings suggest that hospital managers and policy makers should primarily focus on addressing the technical inefficiency issues before examining ways for a possible restructure of their scale of operations. Besides, it should not be overlooked that technical improvement is more controllable and can be addressed in the short-term without requiring the prior change of scale.

Finally, our findings showed that technical and scale efficiencies of all groups were improved at the end of the 5-year period although these were difficult years of the financial crisis. At this point, it is interesting to examine these results against the ordinary indicators used by hospital managers and policy makers. As can be seen in Table 1 for instance, the indicators of average length of stay and occupancy rate have been improved (-18.8% and +6.8% respectively). The same applies to the indicators for medical and other staff resources per inpatient case which were increased by 55% and 47% respectively. Going one step further, we examined all nine possible indicators that can be formed by the ratios of the three outputs when combined with the three inputs (i.e. the six variables that were used in DEA model). Simple comparisons of year’s 2013 values, against those of 2009, showed that eight out of nine indicators significantly improved, the only exception being the (meaningless) ratio of outpatient visits per bed, which was reduced by 10%. Thus, it can be argued that the findings of the study are in line with the whole “macro-picture” depicted in Table 1. The fact that the efficiency improvement between 2009 and 2013, as measured by DEA, is not of the extent implied by the large increment of ordinary indicators can be mainly attributed to its inherent characteristic of relativity in comparisons. The method is capable to perform only relative measurements of efficiency which as such are valid only inside the "borders" of the particular sample. For instance, the improved performance that would be expected by a plain +5% increment throughout all units' outputs, while keeping all their inputs constant, would not be captured by DEA. On the other hand, the overall augmented DEA scores in year 2013, compared with the ones five years before, indicate that eventually some inefficient hospitals in 2009 managed to operate closer to 2013’s efficient frontier defined by their counterpart efficient ones regardless of the relative possible shift and final distance between the two borders which cannot be accounted for by the method. It is evident therefore that DEA findings are complementary to the ones that can (and should) be derived by other means of performance evaluation.

In this context, the productivity of the hospitals between the first (2009) and last (2013) years of the study was also assessed by means of the Malmquist productivity index so that the changes in technology (reflected by shifts in the technology frontier) can be captured as well. Thus, the overall productivity change was subsequently decomposed into technological and efficiency changes whilst the latter was further analyzed into scale and pure technical efficiency changes. The relevant findings were in line with the ones from window analysis as all efficiency related indices were found to be greater than unity meaning that both scale and pure technical efficiency were improved in 2013. As far as the technological change is concerned, the findings revealed progress for the groups of larger hospitals (B and C) and regress for the smaller (groups A and B). The group D with the largest hospitals (>400 beds) experienced the highest technological progress (+20.2%) whilst the group B with the medium to small hospitals (85–190 beds) exhibited the highest regress (-11.1%). On the other hand, the comparisons of efficiency changes showed a twofold improvement for the medium-to-small sized hospitals (groups A+B) compared to the medium-to-large ones (groups C+D). It is therefore deducted that productivity change in larger hospitals is mainly related to changes in technology, as opposed to the smaller ones for which changes in efficiency appears to have higher contribution. It could be argued that the finding is compatible with the fact that larger hospitals are usually equipped with the latest medical technological innovations and personnel and therefore their productivity growth is heavily dependent on them. A similar finding is also reported by Gannon [74] who examined the productivity growth and efficiency in the production of hospital care in Ireland from 1995 to 1998.

Summing up, it can be concluded that there has undoubtedly been a significant performance improvement due to the large increments of outputs and the corresponding simultaneous large decrements in inputs. At the same time, there was also a respectable efficiency improvement meaning that technically the “center of efficiency mass” of the whole system, viewed as a unit, was elevated positively.

The present study has some limitations which should be taken into account. First, patient severity which may influence utilization and therefore measured efficiency has not been incorporated directly in the study. The lack of relevant data did not allow adjusting outputs for variability in case mix across hospitals. This means that efficiency results may be biased since all outputs are considered equivalent across all facilities. Although it is clear that not all of them are equivalent, it can be considered that this limitation has been adequately remedied as all "special" hospitals were excluded from the study whilst the rest of them were further allocated into four fairly homogenous samples in terms of their size and mixture of services they provide (same clinics/specialties). Consequently, the four groups can be considered as consisting of hospitals with similar case mix. Second, it is worth noting that although the technical aspect of efficiency variations has been covered adequately, the equally important efficiency component that relates to quality variations has been left out in this study due to lack of appropriate and reliable data. It is clear that the establishment of a mechanism for collecting more detailed data in a centralized and systematic way will resolve the afore mentioned limitations and undoubtedly improve the completeness and quality of future efficiency studies.

## Conclusion

The fact that health care expenditures represent a substantial proportion of total public expenditure places an enormous pressure to cut costs. Towards this objective in the last five years, the Ministry of Health has an ongoing program of reforms in the public hospital sector. On the other hand, cost containment in the health care sector should be carried out without compromising the quantity and quality of services produced. This is even more pertinent in Greece, given the current economic crisis. One way to compensate for reduced budgets in order to maintain or even improve the level of health care services offered is through identification and elimination of possible sources of inefficiency. It is evident, for instance, that once inefficiency has been eliminated, the saved resources could be devoted to cover other areas of the health care system (improved quality care to patients, innovative technology, staff training). Hence, the establishment of specific and thoroughly researched criteria for measuring efficiency is extremely important. In this context, the present study constitutes an inter-temporal efficiency map for the technical performance of hospital activity in the context of the Greek NHS throughout the five-year period 2009–2013. The empirical findings can be used as a useful guide for both hospital managers and policy makers in their process to improve the health care system, in favor of the true beneficiaries, which are the patients and society in general.
